# SPP1+ Microglia Are Associated with Neuroimmune Rewiring and Glutamatergic Neuronal Injury in ART-Suppressed People Living with HIV

**DOI:** 10.64898/2026.09.04.749241

**Published:** 2026-09-10

**Authors:** Ciniso S. Shabangu, Hongjie Chen, Ashokkumar Manickam, Nikesh Katuwal, Jacob B. Lovins, Edward P. Browne, Sara Gianella, Antoine Chaillon, David M. Margolis, Yuyang Tang, Guochun Jiang

## Abstract

Antiretroviral therapy (ART) effectively suppresses systemic HIV replication but does not eradicate viral reservoirs in the brain, where their identity and contribution to neurological injury remain poorly defined. Using bulk, single-cell, and single-nucleus transcriptomics of postmortem human brain tissue, we identify an activated SPP1⁺ microglial population that expands 5.8-fold during ART and serves as the primary central nervous system (CNS) reservoir, preferentially harboring HIV transcripts. These reservoir microglia adopt a distinct, immune-evasive reprogramming state marked by chronic type I interferon signaling and inflammasome activation, which we recapitulate in primary human microglia via prolonged interferon-β exposure. We show that viral transcription in ART-suppressed brains is dominated by nef, which may sustain the viral reservoir by disrupting host HLA-A and HLA-E presentation machinery. This persistent, immune-evasive state is associated with TREM2-C1QC complement-mediated synaptic pruning and severe DNA damage response dysregulation, culminating in a profound loss of VGLUT1⁺ and GAD67⁺ glutamatergic neurons that persists despite viral suppression. Our findings establish SPP1⁺ microglia as an active, pathogenic CNS reservoir, identifying the SPP1, MHC-I, and complement pathways as therapeutic targets to eliminate viral persistence and reverse HIV-associated neurocognitive dysfunction.

## Full Text Availability

The license terms selected by the author(s) for this preprint version do not permit archiving in PMC. The full text is available from the preprint server.

